# A time bridge for obesity care: protein monitoring adds a timing layer to the EASO pathway while preserving the Lancet Commission definition

**DOI:** 10.1038/s41366-026-02083-6

**Published:** 2026-04-04

**Authors:** Enzo Nisoli, Michele O. Carruba, Alessandra Valerio

**Affiliations:** 1 Center for Study and Research on Obesity, Department of Medical Biotechnology and Translational Medicine, University of Milan, Milan, Italy; 2 Department of Molecular and Translational Medicine, University of Brescia, Brescia, Italy

**Keywords:** Obesity, Obesity

## Abstract

The European Association for the Study of Obesity (EASO) frames obesity care around staged, target-driven decisions. The Lancet Diabetes & Endocrinology Commission provides a disease definition—*clinical obesity* requires objective dysfunction— and distinguishes preventive strategies for preclinical obesity from therapeutic strategies for clinical obesity. Continuous protein monitoring (CPM) refers to wearable or minimally invasive sensors that repeatedly measure short panels of proteins in interstitial fluid or blood and track within-person trends over time. We hypothesize that CPM could help stratify risk and guide the timing of preventive intervention in *preclinical obesity* (excess adiposity, preserved function), and trigger timely escalation within the EASO pathway without changing the Commission’s diagnostic criteria.

## Introduction

Obesity care is shifting from weight-centric snapshots to staged, target-driven decisions. The European Association for the Study of Obesity (EASO) has proposed a pragmatic pathway that diagnoses, stages and intensifies treatment on the basis of structured assessment—not BMI alone—based on an anthropometric entry gate that includes waist-to-height ratio (WtHR ≥ 0.5) and three clinical domains (medical, functional, mental), with predefined targets and escalation rules [1]. In parallel, the Lancet Diabetes & Endocrinology Commission defines clinical obesity as excess adiposity plus objective organ or tissue dysfunction and/or age-adjusted limitations in activities of daily living (ADL), drawing a clear boundary from preclinical obesity, in which function is preserved [2].

**Fig. 1:**
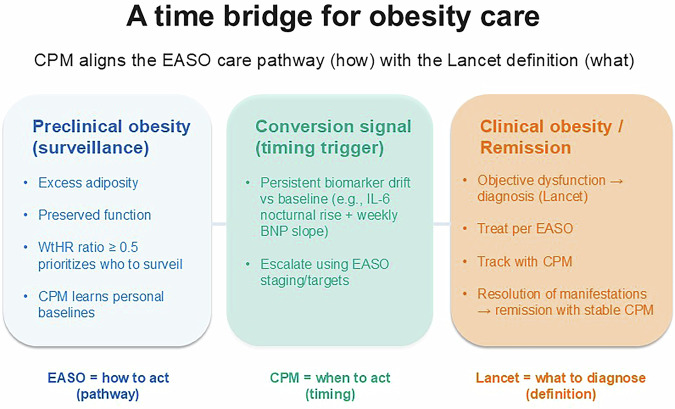
A timing layer for action in preclinical obesity. Individuals with preclinical obesity (excess adiposity, preserved function) enter surveillance based on waist-to-height ratio (WtHR) ≥ 0.5. Continuous protein monitoring (CPM) establishes within-person biomarker baselines and tracks short panels over time. When a conversion signal—a persistent deviation from baseline (for example, nocturnal IL-6 rise with a week-over-week increase in B-type natriuretic peptide, BNP)—is detected, care is escalated along the European Association for the Study of Obesity (EASO) pathway. Clinical obesity is diagnosed only when objective dysfunction is present, per the Lancet Diabetes & Endocrinology Commission; resolution of manifestations indicates remission. CPM is proposed to support timing of preventive intervention within preclinical obesity; diagnosis of clinical obesity remains based on objective dysfunction per the Lancet Commission. WtHR waist-to-height ratio, CPM continuous protein monitoring, BNP B-type natriuretic peptide, NT-proBNP N-terminal pro–B-type natriuretic peptide.

These frameworks are distinct by design. EASO provides a diagnostic–therapeutic pathway for staging burden and guiding escalation, whereas the Commission provides a disease definition that separates preclinical from clinical obesity and distinguishes preventive from therapeutic goals. A practical challenge is timing—deciding when, within preclinical obesity, to intensify preventive care for individuals whose course suggests near-term progression. Continuous protein monitoring (CPM) refers to wearable or minimally invasive sensors that repeatedly measure short panels of proteins in interstitial fluid or blood and track within-person trends over time. In our hypothesis, CPM is used to establish an individual baseline and then to detect persistent deviations (“conversion signals”) that may justify earlier escalation within the EASO pathway, while leaving the Commission’s diagnostic threshold unchanged [3] (Fig. 1).

## What each proposal gets exactly right

The EASO framework works in routine care because it resembles modern chronic-disease management. It begins with simple anthropometry—especially WtHR to capture central adiposity—then requires a structured appraisal across medical, functional, and mental domains, sets patient-centered targets, and intensifies when goals are not met [1, 4]. This approach reduces therapeutic inertia by providing clear next steps and by prioritizing outcomes that matter (function, quality of life, and complication control) alongside weight.

The Lancet Commission strengthens the disease definition. By separating preclinical from clinical obesity and requiring objective dysfunction (or age-adjusted ADL limitations) for disease status, it helps prevent overdiagnosis, anchors remission to the resolution of clinical manifestations rather than to kilograms lost, and clarifies when a diagnosis should trigger access and coverage [2]. Importantly, the Commission does not argue against early action; it explicitly distinguishes preventive strategies for preclinical obesity from therapeutic strategies for clinical obesity. In this framing, the Lancet Commission is not a rival pathway and EASO is not an alternative diagnosis. One is a definition of illness—a line crossed only when objective dysfunction is present; the other is a care pathway—a way to stage burden, set targets, and escalate treatment. Keeping them distinct preserves diagnostic specificity and avoids overdiagnosis; using them together makes care timely and accountable [1, 2].

## What continuous protein monitoring adds

**Fig. 2:**
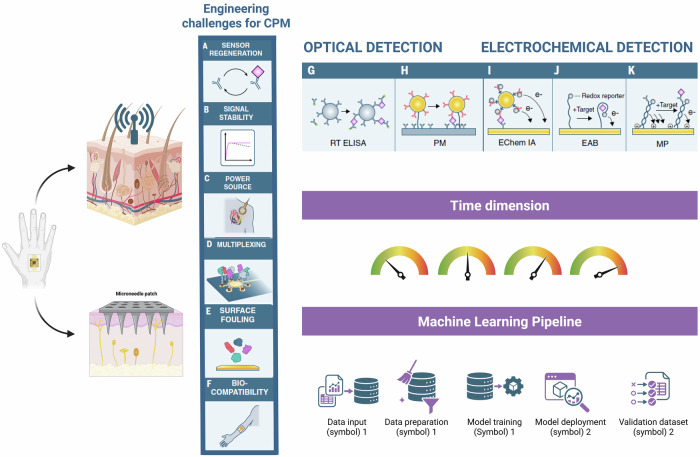
Engineering roadmap for continuous protein monitoring. Left, representative skin-interfacing form factors (a subdermal/wearable module and an adhesive microneedle patch) sampling interstitial fluid. Center, key engineering challenges (A–F): sensor regeneration (repeated binding cycles), signal stability/drift, power/telemetry, multiplexing of small panels, surface/biofouling, and biocompatibility. Top right, detection strategies: Optical—real-time ELISA (RT-ELISA) and plasmonic modalities (PM); Electrochemical—electrochemical immunoassays (EChem-IA), electrochemical aptamer-based sensors (EAB) and the molecular pendulum (MP) approach. The Time dimension highlights that actionable sensing requires resolving changes over minutes–days; gauges emphasize trend-level readouts rather than single snapshots. Bottom, an illustrative machine-learning pipeline (data input, preparation, model training, deployment, and validation) for converting streams into alerts. CPM continuous protein monitoring, RT-ELISA real-time ELISA, PM plasmonic modalities, EChem-IA electrochemical immunoassay, EAB electrochemical aptamer-based, MP molecular pendulum. Adapted from Donnelly et al. [3]). The figure was created with BioRender.com.

CPM is not proposed to reconcile frameworks, but to add longitudinal timing information that could help decide when to intensify preventive intervention within preclinical obesity. Progression from risk to illness is temporal. CPM restores that missing dimension without redefining disease. Advances in reagentless, reset-capable affinity sensors (including molecular-pendulum and electrochemical aptamer-based designs) have enabled near–real-time, in-vivo tracking of short protein panels that report on inflammatory tone, adipose–gut–brain signaling, and cardiometabolic load (Fig. 2) [3, 4]. The analogy with continuous glucose monitoring (CGM) is instructive: CGM improved outcomes not by renaming hyperglycemia, but by converting snapshots into streams that supported earlier, more consistent decisions [5]. Cardiology offers another precedent: high-sensitivity troponin compressed emergency pathways with time-based algorithms, and natriuretic peptides track cardiac load and help anticipate decompensation [6, 7]. CPM applies the same logic focused on timing to obesity medicine (Fig. 2).

Two features make CPM clinically usable rather than merely technological. First, within-person baselines: rather than comparing a patient to population cutoffs, CPM spends a short calibration phase learning that individual’s signal range and circadian structure (for example, higher evening IL-6 with a predictable nocturnal dip) and then tracks deviations from that learned pattern. This is crucial in obesity, where inter-individual variability is high, trajectories are heterogeneous and comorbidities common; a sustained percentage change from one’s personal baseline—defined using the reference change value (RCV)—can be more informative than crossing a static threshold [8]. Second, cadence matching: sample proteins at biologically sensible intervals—minutes to hours for cytokines and appetite hormones and daily or weekly for cardiac stress markers—so the data are dense enough to inform decisions without adding noise [7, 9–12]. Together, these choices keep CPM interpretable (simple rules relative to a known baseline) and actionable (alerts tied to a time window clinicians can respond to).

Beyond inflammatory tone and cardiometabolic load, short panels can also capture behaviorally relevant physiology. Shifts in ghrelin amplitude or disrupted glucagon-like peptide-1 (GLP-1)/glucose-dependent insulinotropic polypeptide (GIP) rhythms around meals can indicate deteriorating appetite control or treatment non-response; a flattening of adiponectin dynamics may signal worsening adipose tissue health. None of these signals redefines disease; they anticipate the clinical picture that the Lancet Commission requires for diagnosis, buying time to intensify care through the EASO pathway.

## A practical synthesis clinicians and payers can live with

In practice, CPM lets clinicians treat preclinical obesity as a monitored, intervenable state, consistent with the EASO pathway and respectful of the Commission’s boundary. We do not propose harmonizing definitions; rather, CPM is a hypothesis for timing preventive escalation within preclinical obesity, while diagnosis of clinical obesity remains anchored to objective dysfunction. Individuals who meet anthropometric entry criteria—WtHR ≥ 0.5, a simple, ethnicity-agnostic gate endorsed internationally—begin a short calibration period to establish within-person biomarker baselines [1, 4]. Thereafter, care teams watch for conversion signals: transparent, pre-specified patterns—for example, a sustained nocturnal rise in IL-6 accompanied by a week-over-week increase in B-type natriuretic peptide (BNP) or N-terminal pro–B-type natriuretic peptide (NT-proBNP)—that indicate near-term progression and warrant action [7, 9–11]. When such a signal persists, clinicians escalate along the EASO pathway—tightening behavioral therapy, initiating or up-titrating anti-obesity medications, or evaluating device/procedural options—before dysfunction becomes established [1, 13]. If objective dysfunction emerges, the Commission’s criteria are met and clinical obesity is diagnosed; if not, the patient still receives timely, proportionate intervention rather than passive observation.

## Comparators and incremental utility

CPM is not proposed as a replacement for established risk tools. Any clinical value must be demonstrated on top of readily available factors, including family history, anthropometrics (beyond BMI, including waist-to-height ratio), and standard metabolic parameters. We therefore frame CPM as a hypothesis for improving timing and consistency of action—not as a proven predictor of progression—and propose that its utility should be judged by incremental prediction and clinical impact relative to these comparators (for example, time to first diagnosis of clinical obesity per Commission criteria, patient-reported function, quality of life, and health-care utilization). Crucially, studies should quantify incremental utility beyond readily available comparators (family history, anthropometrics including waist-to-height ratio, and standard metabolic parameters), rather than treating biomarker streaming as inherently superior.

This synthesis fits the current therapeutic landscape. A recent Nature Medicine network meta-analysis led by EASO investigators—56 trials and more than 60,000 participants—confirms double-digit mean weight loss with semaglutide and tirzepatide and shows signals of benefit across selected complications, supporting earlier, targeted escalation when credible early-warning signals arise [13]. Notably, an EASO-aligned approach supports broad eligibility for anti-obesity pharmacotherapy, beyond narrow thresholds based solely on functional impact [13]. Importantly, our argument is not that CPM is needed to justify access to pharmacotherapy within an EASO-aligned approach. Rather, CPM is proposed as a hypothesis to improve timing, targeting, and longitudinal monitoring in real-world care—helping distinguish trajectories that are actively deteriorating from those that are relatively stable, supporting treatment adjustment, and informing de-intensification during remission surveillance. The definition–pathway complementarity avoids a false choice between sensitivity and specificity: EASO governs how and when we act; the Commission governs what we diagnose.

Two brief vignettes illustrate the flow. Case 1 (central adiposity, preserved function): a 49-year-old woman (BMI 27; waist-to-height ratio, WtHR 0.57) reports fatigue but has normal echocardiography and liver elastography. She enters surveillance. After a two-week calibration, CPM shows a persistent nocturnal IL-6 rise and a week-over-week increase in BNP over six weeks. She has not met clinical-obesity criteria, but the conversion signal is positive. The team escalates per EASO: structured behavioral therapy refocused on sleep and meal timing, initiation of a GLP-1–based medication, and close follow-up. Three months later, symptoms improve, the biomarker pattern normalizes, and she remains preclinical—a course likely altered by earlier action. Case 2 (remission tracking): a 58-year-old man with clinical obesity and heart failure with preserved ejection fraction (HFpEF) undergoes intensive therapy (lifestyle plus pharmacotherapy). Function improves; on reassessment he no longer meets the Commission’s dysfunction criteria. CPM then documents stable, low-variance cytokine and BNP patterns for 12 weeks, supporting a decision to de-intensify while maintaining surveillance for relapse [2, 5, 7].

For payers, the synthesis is cleaner: EASO governs how and when we act, the Commission governs what we diagnose, and CPM supplies an auditable timing signal tied to outcomes that matter—time to clinical-obesity diagnosis, quality of life, functional capacity, and complication control [1–3]. Coverage can align with this logic: support CPM when it reduces conversions to clinical obesity or improves sustained remission; support therapy escalation when a predefined conversion signal is present; and grant full disease benefits when objective dysfunction is documented.

## What would change on the ground

Three practical shifts follow immediately. First, case finding becomes sharper and fairer: WtHR ≥0.5 flags whom to surveil, and CPM then distinguishes stable risk from active drift [1, 4]. Second, decisions become less tied to appointment timing and more signal-driven, reducing unwarranted variation across clinics and regions [1, 5]. Third, remission becomes more robust: alongside restored function, low-variance biomarker stability provides an auditable signature of control over time—useful for de-intensification decisions and payer dialogue [2, 3].

Because CPM yields continuous rather than episodic data, clinics can move away from escalation gated by the accident of visit timing. Teams can batch-review weekly CPM dashboards alongside EASO targets, focusing care where signals are drifting while keeping stable patients on lower-intensity follow-up. For patients, this feels less like “wait until you worsen” and more like proactive course correction. For health systems, it enables capacity planning (matching resources to rising-risk panels) and learning-health loops in which rules are refined and pre-registered as signals and outcomes accumulate [1, 5].

## What needs testing (and how to keep this safe and fair)

The research path is pragmatic. Panel composition and sampling cadence should be pre-registered and narrow, with predefined performance targets for sensor stability, baseline-drift correction, and alert burden. Key risks include non-specific signals confounded by comorbidities, as well as false-positive and false-negative alerts that could drive over- or undertreatment. Conversion signals must be interpretable, easy to explain to patients, and prospectively tied to outcomes that matter—time to first diagnosis of clinical obesity, quality of life, functional status, and complication rates—mirroring the clarity achieved with high-sensitivity troponin algorithms and natriuretic-peptide monitoring in cardiology [6, 7]. Randomized pragmatic trials in adults with BMI ≥ 25 and WtHR ≥ 0.5 should compare CPM-guided intensification versus standard EASO care; in parallel, implementation studies should address privacy-by-design, data minimization, electronic health record (EHR) integration, and equity, so that streaming data reduces—rather than widens—disparities (Fig. 1) [14, 15]. Framed as secondary prevention in preclinical obesity, CPM can follow the CGM precedent for coverage, with professional societies setting reporting standards (sensor stability, alert ceilings, positive predictive value) and ethical guardrails for data rights and algorithm transparency (Fig. 2) [12, 15].

Three trial layers are feasible. First, algorithm-calibration studies should enroll adults with BMI ≥ 25 and WtHR ≥ 0.5 to learn baseline variability, define 2–3 candidate conversion signals, and set alert ceilings (for example, ≤ 0.2 alerts per patient-month). Second, pragmatic randomized controlled trials (RCTs) should compare CPM-guided care with standard EASO care, using time to first diagnosis of clinical obesity (per Lancet Commission criteria) as the primary endpoint, with secondary endpoints that include quality of life, patient-reported function, cardiometabolic events, and health-care utilization [1–3, 13]. Third, implementation/equity studies should test data-minimization defaults, patient-facing explanations, multilingual literacy supports, and device-access programs to avoid a digital divide [14, 15]. Across all layers, pre-registration of panels, alert rules, and performance targets (stability, drift correction, positive predictive value) should be standard, with society guidance on privacy and data rights following the digital-health playbooks already in use [14, 15].

A practical reporting and implementation checklist for researchers can reduce heterogeneity across CPM studies and ensure that results can be compared, reproduced, and appropriately critiqued: (i) panel composition and sampling cadence; (ii) the baseline-learning period; (iii) the alert rule (inputs, time window, required persistence, and action); (iv) alert-burden metrics; (v) linkage to outcomes; and (vi) equity and access measures (device provision, language support, cost coverage). Used consistently, these elements make CPM studies easier to compare across cohorts and platforms and help distinguish signal from noise. None of this changes the diagnostic line; it simply supports transparent timing rules for earlier action within the EASO pathway while reserving the disease label for patients who meet the Lancet Commission’s definition.

## Conclusion

Obesity care is best served by pairing two ideas that are distinct yet complementary. The EASO pathway offers a staged, target-driven way to act; the Lancet Diabetes & Endocrinology Commission supplies a high-specificity disease definition in which clinical obesity requires objective dysfunction. CPM adds the missing dimension of time: short, clinically curated protein panels tracked as within-person trends can surface conversion signals that prompt earlier EASO-style escalation while reserving the diagnostic label for those who meet the Commission’s criteria. What the field needs next are proof and guardrails: narrow panels, transparent rules linked to patient-relevant endpoints, pragmatic trials against standard EASO care, and implementation standards that secure privacy and equity. The goal is simple and shared: act sooner, measure better, and diagnose only when disease is objectively present.
